# Gallium nitride-based geometric and propagation metasurfaces for vortex beam emissions

**DOI:** 10.1016/j.heliyon.2024.e25436

**Published:** 2024-01-27

**Authors:** Meng-Hsin Chen, Yan-Liang Liu, Vin-Cent Su

**Affiliations:** Department of Electrical Engineering, National United University, Miaoli 36003, Taiwan

## Abstract

This work experimentally demonstrates the highly-efficient geometric and propagation metasurfaces for vortex beam emissions. These metasurfaces are respectively composed of high-aspect-ratio fin-like and cylindrical gallium nitride (GaN) meta-atoms. Remarkably, the optimized configuration of the fin-like GaN meta-atoms achieves a cross-polarization transmission efficiency of up to 99 %. Similarly, the cylindrical GaN meta-atoms exhibit an average co-polarization transmission efficiency of 97 %. Both metasurfaces, designed for vortex beam emission, exhibit annular intensity converging capabilities at distinct wavelengths in the visible. Notably, the geometric metasurface shows achromatic annular intensity distributions over a continuous wavelength range up to 100 nm, in sharp contrast to the propagation metasurface, which is subject to inherent wavelength dispersion limitations.

## 1 Introduction

Optical vortices [1,2] propagate in the paraxial approximation, imparting a distinctive orbital angular momentum (OAM) [3] to give rise to optical vortex beams (OVBs) [[4], [5], [6], [7]]. Such beams [8] harness the inherent phase dependence in the azimuthal form of $e^{im\varphi }$, where m denotes the well-established topological charges (TCs). This feature extends to a range of values, resulting in OVBs with a wide variety of topological charges, leading to an angular momentum per photon of $\frac{h}{2\pi }m$. The realm of OVBs has witnessed a diverse spectrum of applications, including particle manipulation [9,10], optical communication [11,12], quantum information processing [13,14], and non-linear opitcs [15]. Nonetheless, conventional methodologies typically rely on the utilization of cumbersome optical components. Examples include diffraction gratings [16,17], spatial light modulators [18,19], and spiral phase plates [20], which significantly constrain the potential for broader deployment of OVBs, particularly in the context of planar optics. Furthermore, the applicability of these techniques within the semiconductor industry remains severely limited, posing challenges to their integration into mainstream manufacturing processes.

Positioned at the foresight of future optics, metasurfaces have emerged as a fascinating technological frontier for the realization of compact devices capable of semiconductor-compatible fabrication techniques. Operating in accordance with the principle of the generalized law [21,22] governing light refraction and reflection, metasurfaces are artificially composed of meta-atoms spaced in sub-wavelength periods. This intentional configuration performs pronounced phase discontinuities at the interface between two mediums. Therefore, diverse anomalous optical phenomena have been explored by these metasurfaces made of various materials [[23], [24], [25]], thereby realizing potential across versatile applications [[26], [27], [28], [29], [30], [31], [32], [33], [34], [35], [36], [37], [38], [39], [40], [41]]. Among these notable achievements, wide bandgap gallium nitride (GaN), one of the third-generation semiconductors, is characterized by its remarkable ability to exhibit high refractive index contrast across the visible spectrum. This intrinsic property positions GaN as an ideal candidate for being one of the high-contrast metasurfaces [22,42]. Together with double-side polished sapphire as substrates, meta-atoms formed with high-aspect-ratio GaN have performed a superior device performance. Particularly noteworthy is their current research and application status that are focusing on the realization of metalenses [[43], [44], [45], [46], [47]], capable of diffraction-limited focusing. Moreover, there are many works on metasurfaces made of meta-atoms with various materials for focused vortex beam generation such as gap-surface plasmonic metals [48], rectangular-hole gold antenna [49], elliptical cylinder antenna [50], cylindrical silicon nitride [51], rectangular [27] and cylindrical [28] GaN nanopillars, and so on. Nevertheless, comprehensive comparative studies of highly efficient GaN metasurfaces designed using geometric (Pancharatnam-Berry) and propagation phase principles for vortex beam emissions remain an understudied area, warranting further investigation and scholarly attention.

In this work, we successfully demonstrated exceptionally efficient geometric and propagation metasurfaces utilizing high-aspect-ratio GaN meta-atoms for OVB emissions at visible wavelengths. The optimized meta-atoms have been rigorously engineered using commercially available software CST. Through meticulous experiments, we have comprehensively characterized the converging capabilities of these metasurfaces at distinct wavelengths in the visible. Furthermore, a laser beam capable of a tunable center wavelength with a bandwidth of 100 nm in the visible has been selected as incident light to illuminate the metasurfaces. The utilization of this laser allows us to gain deeper insights into the wavelength dispersion mechanism—chromatic aberration—inherent to both metasurfaces.

## 2 Simulation results

The phase retardation profile essential for the metasurfaces to function as OVBs can be expressed as:

$$\varphi \left(x,y\right)=m\times \tan^{-1}\;\left(\frac{y}{x}\right)+\frac{2\pi }{\lambda }\left[\left(f-\sqrt{{(x}^{2}+y^{2})+f^{2}}\right)\right]$$

**Fig. 1:**
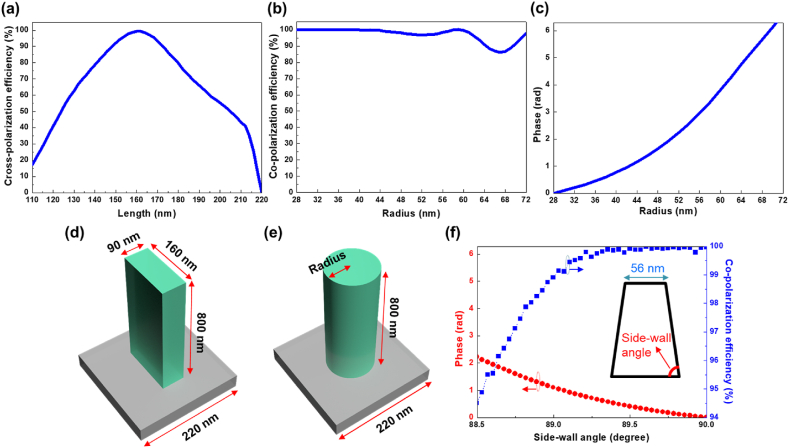
The simulation results for (a) cross-polarization efficiency in relation to various lengths at the width of 90 nm for the geometric phase design. (b) and (c) are respectively the co-polarization efficiency and the phase versus different radii of the cylindrical structures for the propagation phase design. (d) and (e) correspond to the schematics for fin-like and cylindrical meta-atoms. (f) The phase and co-polarization efficiency in relation to the side-wall angle.

Here, the variable m denotes the TCs, while x and y represent the Cartesian coordinates, λ signifies the free space wavelength, and f corresponds to the focal distance. The increase in the variable m results in the appearance of the OVBs with larger diameters of the ring-shaped light intensities. Geometric and propagation metasurfaces are both configured at a wavelength of 450 nm, a TC of 6, a diameter of 100 μm, and a focal distance of 150 μm. The mechanism for full phase control of geometric metasurfaces depends on rotating the direction of meta-atoms with identical dimensions. The optimal parameters are achieved for fin-shaped meta-atoms, possessing a width of 90 nm, a length of 160 nm, and a sub-wavelength period of 220 nm, leading to an impressive cross-polarization transmission efficiency as high as 99 % at the designed wavelength of 450 nm, as illustrated in Fig. 1(a). Conversely, the propagation metasurfaces rely on an alternate strategy by adjusting the geometry of cylindrical meta-atoms rather than rotation. This geometry modification in this study exploits the change in cylinder diameter to facilitate 2π phase modulation, as revealed in Fig. 1(b) and (c). The simulated results demonstrate an average co-polarization transmission efficiency of up to 97 % for cylindrical meta-atoms with radii ranging from 28 to 72 nm in a sub-wavelength period of 220 nm at the incident wavelength of 450 nm. The schematics for the unit cells of the fin-like and cylindrical GaN meta-atoms with associated feature sizes can be found in Fig. 1(d) and (e). We also note that the tilted sidewalls and uneven widths make the phase deviate from the desired phase and reduce the transmission amplitude. Therefore, we have taken the effect of the sidewall angle in the simulation as shown in Fig. 1(f). A profound deviation from the desired phase and transmission amplitude can be discovered in the figure as the tilted sidewalls have been observed.

## 3 Experimental results

**Fig. 2:**
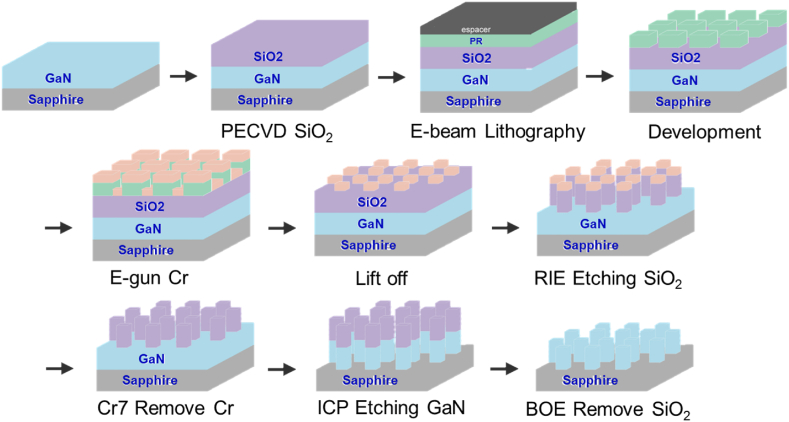
The Fabrication processes for high-aspect-ratio GaN meta-atoms for the construction of the geometric and propagation metasurfaces.

The fabrication process was executed, as outlined in Fig. 2. Initially, an un-doped GaN epitaxial layer, with a thickness of 800 nm, was grown on a double-polished sapphire substrate via metal-organic chemical vapor deposition (MOCVD). Subsequently, a SiO_2_ layer was deposited onto the GaN epi-substrate, followed by a spin-coated resist layer on it. Employing the E-beam lithography system, a concentrated electron beam was exposed onto the top surface of the resist-coated substrate. Following this exposure, a 30-nm-thick layer of chromium (Cr) was evaporated onto the developed resist-coated substrate. After the lift-off process, reactive-ion etching (RIE) and inductively coupled plasma reactive ion etching (ICP-RIE) machines were used to fabricate these high-aspect-ratio GaN meta-atoms. The final step employed a buffered oxide etch (BOE) solution to remove residual SiO_2_.

**Fig. 3:**
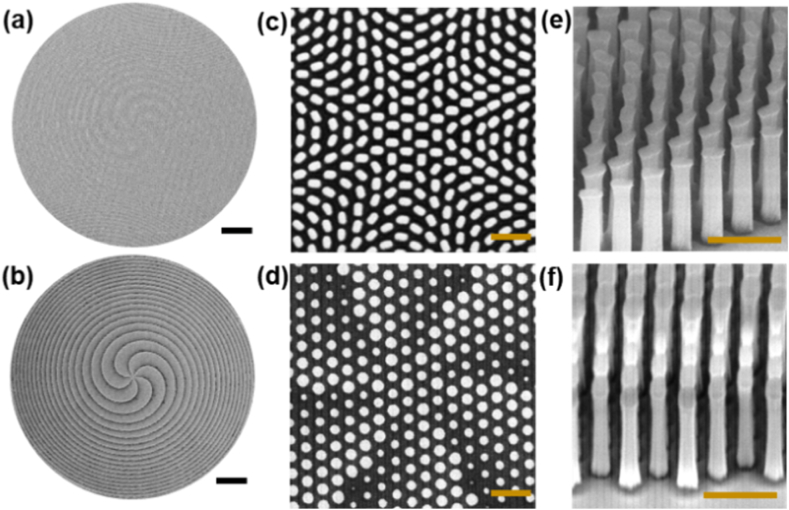
SEM images for top views of the metasurfaces with (a) the geometric design, (b) the propagation design. The zoom-in top- and tilt-view SEM images of (c, d) the center, (e, f) the edge, respectively, for geometric and propagation samples. Scale bar, 10 μm in Fig. 3(a) and (b). Scale bar, 500 nm in Fig. 3(c)-3(f).

The top-view scanning electron microscope (SEM) images of the fabricated samples based on the geometric and propagation design principles are presented in Fig. 3(a) and (b), respectively. The corresponding close-ups of the central regions are shown in Fig. 3(c) and (d). The recognizable fin- and cylinder-shaped meta-atoms are evident within these images, illustrating the distinctive design principles for each metasurface configuration. The edge tilt-view SEM depictions featured in Fig. 3(e) and (f) for the geometric and propagation metasurfaces, respectively, exhibit a vertically inclined sidewall morphology with surface smoothness. We note that it is imperative to emphasize the extraordinary complexity associated with the fabrication of *meta*-GaN atoms characterized by near-vertical sidewalls and high aspect ratios.

**Fig. 4:**
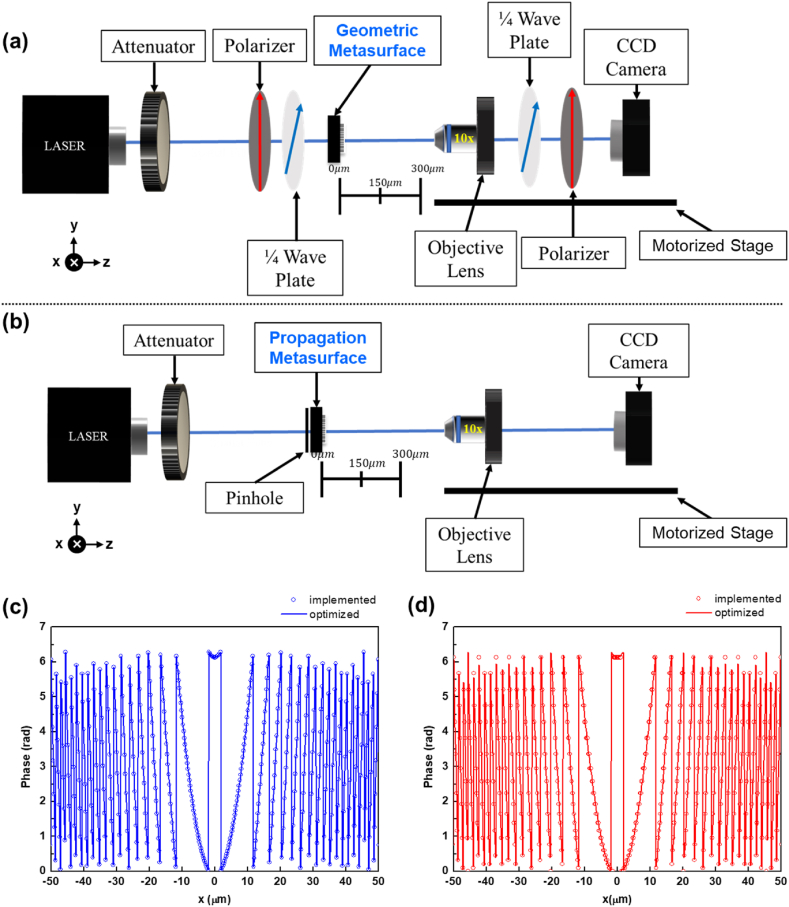
The experimental setup for measuring the annular intensity profiles for (a) the geometric metasurface, (b) the propagation metasurface. (c) and (d) respectively illustrate the phase distributions at the center y-axis position for the geometric and propagation metasurfaces with solid lines as theoretically optimized phase profiles and hollow holes as the implemented phase distributions.

The optical setup employed to capture the annular intensity of the geometric metasurface is depicted in Fig. 4(a). Herein, a supercontinuum white laser with a tunable filter based on acousto-optic tunable filter technology (AOTF) generates laser beams of different wavelengths as incident light, sequentially propagating through an attenuator, a polarizer, and a quarter wave plate. The propagation direction of the laser beams is along the z-axis. This arrangement yields a circularly polarized light for illuminating the geometric metasurface. The emission light from the metasurface is captured via a 10× objective lens positioned on a motorized stage. Within this apparatus, the polarizer and the quarter-wave plate on the motorized stage are used to filter out undesired environmental light. Fig. 4(b) illustrates the optical setup tailored for the investigation of the propagation metasurface, akin to its counterpart in Fig. 4(a). Diverging from the aforementioned configuration, a precision-engineered pinhole is placed in front of the metasurfaces without the inclusion of polarizers and quarter-wave plates. Such optical arrangement is based on the utilization of a linearly polarized incident laser. Moreover, the theoretically optimized and implemented phase distributions at the center y-axis position in relation to the x-axis direction are respectively shown in Fig. 4 (c) and 4 (d). The results show that the implemented phase distributions are extremely close to the idea phase requirements.

**Fig. 5:**
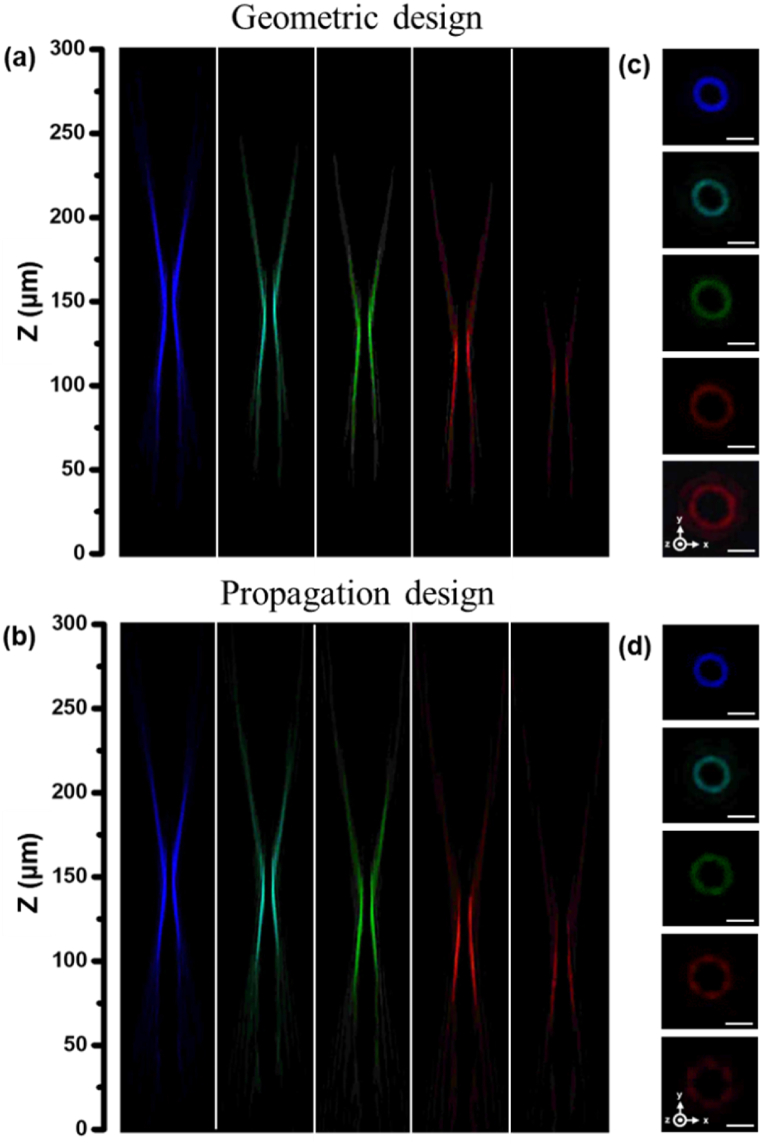
The annular intensity distributions in the x-z plane at the wavelengths of 450, 500, 550, 600, and 650 nm for (a) the geometric device, (b) the propagation device, respectively. The intensity distributions in the x-y plane at the wavelengths of 450, 500, 550, 600, and 650 nm for (c) the geometric device, (d) the propagation device, respectively. Scale bar, 5 μm in Fig. 5(c) and (d).

Fig. 5(a) and (b) reveal the outcome of experimental measurements, distinctively illustrating the performance of both the geometric and propagation metasurfaces across discrete wavelengths—namely, 450, 500, 550, 600, and 650 nm. Notably, both sets of results demonstrate the annular light distributions achieving precise convergence, consistently aligning with the designated focal distance of 150 μm at the 450-nm wavelength. Such annular light converging phenomenon persists across the spectrum for both metasurfaces, firmly establishing their strength in realizing convergence capabilities at distinct wavelengths in the visible. The experimentally measured transmission efficiencies at different incident wavelengths for the geometric- and propagation-phase design metasurfaces can be found in Table 1, demonstrating the high transmission efficiencies achievable in the visible. The results of mode purity [52] for the geometric- and propagation-phase design metasurfaces at the operating wavelength of 450 nm have been respectively measured as high as 99 % and 76 %, showing a successful fabrication of the proposed metasurfaces at the design wavelength.

**Table 1:** The experiment results of the transmission efficiency at different incident wavelengths.

Incident Wavelength (nm)	450	500	550	600	650
Geometric design	80 %	56 %	42 %	20 %	92 %
Propagation design	86 %	91 %	89 %	84 %	87 %

Furthermore, the investigation reveals deeper insights into the convergence behavior as wavelengths extend. The convergence of annular light occurs at progressively smaller z-axis coordinates as the wavelength lengthens. This phenomenon inherently discovers the anomalous properties of metasurfaces. The diameters of the ring-shaped light intensities observed at these converging z-axis positions also exhibit an increase in correspondence with larger wavelengths. This phenomenon is depicted in Fig. 5(c) and (d), thoughtfully associated with the geometric and propagation samples, respectively.

**Fig. 6:**
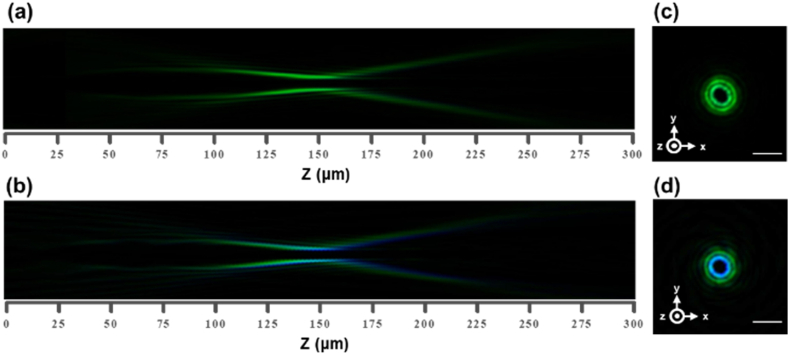
The annular intensity distributions in the x-z plane measured at the center wavelength of 500 nm across a continuous wavelength region from 450 to 550 nm for (a) the geometric metasurface, (b) the propagation metasurface. The corresponding annular intensity distributions in the x-y plane at the z-axis position of 157 μm for (c) the geometric metasurface, (d) the propagation metasurface. Scale bar, 5 μm in Fig. 6(c) and (d).

However, the achromatic capability of the metasurface design based on geometric phase principles is superior to those that achieved through the propagation phase method. This is because the design principle for the geometric phase method is based on the rotation of identical asymmetric meta-atoms to achieve the desired phase distribution, and such rotation behavior is insensitive to wavelength dispersion constraints. However, the propagation phase design principle is to modify the morphology of the meta-atoms to acquire the intended phase profile. These morphology modifications of the meta-atoms always lead to the abrupt phase contribution varying with the different incident wavelengths, making responses frequency-sensitive. To meticulously validate this intrinsic achromatic capability, the supercontinuum white laser is equipped with a center wavelength tunable filter with a bandwidth of 100 nm in the visible. Fig. 6(a) and (b) exhibit the annular intensity profiles in the x-z plane, centered around the 500 nm wavelength, with the bandwidth maintained at 100 nm. The results show the geometric metasurface maintaining remarkable achromatic performance across a continuous wavelength region from 450 to 550 nm, significantly better than its propagation phase counterpart. Further insight into this comparison is realized through the images in the x-y plane at the z-axis position of 157 μm slightly beyond the converging distance, meticulously depicted in Fig. 6(c) and (d) for the geometric and propagation devices, respectively. Impressively, while Fig. 6(d) clearly exhibits pronounced and deleterious tendency to chromatic aberration, the geometric metasurface displays remarkable chromatic-free results, showing its commendable achromatic capability.

## 4 Conclusion

In this work, we have successfully demonstrated the geometric and propagation metasurfaces for vortex beam emissions. These metasurfaces are composed of high-aspect-ratio GaN meta-atoms in fin-like or cylindrical configurations. The simulated cross-polarization transmission efficiency for the optimized fin-like meta-atom shows an exceptionally high value of up to 99 %. The propagation metasurface possesses a 97 % average co-polarization transmission efficiency among the cylindrical radii from 28 to 72 nm. Both kinds of metasurfaces preserve excellent annular intensity converging capabilities at distinct wavelengths at visible but perform shorter converging distances with an increase in wavelengths. Moreover, the geometric metasurface exhibits achromatic annular intensity profiles over a continuous wavelength region from 450 to 550 nm. In contrast, a strong chromatic aberration appears for the metasurface designed with the propagation principle.

## Data availability statement

Data will be made available on request.

## CRediT authorship contribution statement

**Meng-Hsin Chen:** Writing – review & editing, Writing – original draft, Visualization, Validation, Supervision, Software, Resources, Project administration, Methodology, Investigation, Funding acquisition, Formal analysis, Data curation, Conceptualization. **Yan-Liang Liu:** Validation, Methodology, Data curation. **Vin-Cent Su:** Writing – review & editing, Writing – original draft, Visualization, Validation, Supervision, Resources, Project administration, Methodology, Investigation, Funding acquisition, Formal analysis, Data curation, Conceptualization.

## Declaration of competing interest

The authors declare that they have no known competing financial interests or personal relationships that could have appeared to influence the work reported in this paper.
